# Closing the knowledge gap: identifying research priorities for firearm-related injury and mortality in Canada

**DOI:** 10.24095/hpcdp.46.1.01

**Published:** 2026-01

**Authors:** Lotus Alphonsus, Anne Sorvari, Alexa R. Yakubovich, Carmen Gill, Annette Bailey, Carolyn Snider, Wendy Cukier, Irvin Waller, Wendy Thompson, Stephanie Toigo, Nancy Baxter, R. Blake Brown, Natasha Saunders, David Gomez

**Affiliations:** 1 Temerty Faculty of Medicine, University of Toronto, Toronto, Ontario, Canada; 2 Li Ka Shing Knowledge Institute, St. Michael’s Hospital, Toronto, Ontario, Canada; 3 Department of Community Health and Epidemiology, Dalhousie University, Halifax, Nova Scotia, Canada; 4 Department of Sociology, University of New Brunswick, Fredericton, New Brunswick, Canada; 5 Faculty of Community Services, Toronto Metropolitan University, Toronto, Ontario, Canada; 6 Unity Health Toronto, St. Michael’s Hospital, Toronto, Ontario, Canada; 7 Department of Entrepreneurship and Strategy, Toronto Metropolitan University, Toronto, Ontario, Canada; 8 Institute of Health Policy, Management and Evaluation, University of Toronto, Toronto, Ontario, Canada; 9 Department of Criminology, University of Ottawa, Ottawa, Ontario, Canada; 10 Centre for Surveillance and Applied Research, Public Health Agency of Canada, Ottawa, Ontario, Canada; 11 Faculty of Medicine and Health, University of Sydney, Sydney, Australia; 12 Department of History, Saint Mary’s University, Halifax, Nova Scotia, Canada; 13 The Hospital for Sick Children, Toronto, Ontario, Canada; 14 Department of Paediatrics, University of Toronto, Toronto, Ontario, Canada; 15 ICES, Toronto, Ontario, Canada

**Keywords:** firearms, gun violence, injury prevention, public health, priority-setting

## Abstract

**Introduction:**

Firearm-related injury and death are leading yet preventable causes of premature death in Canada. Our objective was to identify knowledge gaps and research priorities to inform a national research agenda to prevent firearm-related injury and death.

**Methods:**

In a two-stage process, nominal group technique was used to encourage experts in firearm injury and death (N = 15) to generate ideas relevant to knowledge gaps in three areas: unintentional firearm injury, intimate partner violence (IPV)/femicide and other firearm-related assaults. Relevant parties (N = 43) subsequently voted on the identified gaps to determine top priorities for future research.

**Results:**

In Stage 1, the experts identified 22 knowledge gaps in unintentional firearm injury, 16 in IPV-related firearm injury/femicide and 33 in other assault-related firearm injuries. Based on their importance and feasibility as research projects, they then selected five, three and seven, respectively, of these knowledge gaps. In Stage 2, the top priorities for future research emerged: the economic cost of firearm injuries to victims’ families and communities and Canadian society; the impact of social policies and legislation aimed at reducing IPV/femicide-related firearm injuries and deaths; and a description of the available and required Canadian firearm-injury data.

**Conclusion:**

The top priorities highlight the large and diverse gaps in knowledge about firearm injury and death in Canada. This marks the first step toward developing a national research agenda for firearm-related injuries. Next steps include operationalizing these gaps into research questions, identifying data sources and methodological approaches, and choosing knowledge translation strategies.

**Highlights**

- Experts identified knowledge gaps to inform a research agenda that focuses on preventing firearmrelated injury and death in Canada.
- There is a lack of accessible and usable data on unintentional and intentional firearm injuries in Canada.
- Researching the economic cost of firearm injuries to victims’ families and communities and Canadian society was identified as the top priority.
- The barriers to collecting firearminjury data and the impact of social policies and legislation to reduce intimate partner firearm injury and femicide were also identified as key priorities for research.

## Introduction

Firearm-related injury is a leading yet preventable cause of premature death in Canada.^1^ Canada ranks third among the G7 countries in terms of age-adjusted firearm-related mortality.^2^ Firearm-related violent crime increased by 55% between 2013 and 2022 (23.7 to 36.7 incidents per 100000 population),^3^ and the homicide rate in 2022 was 2.24 per 100000 population, the highest in 30 years.^1^ More than one in three homicides (41% in 2022) are firearm related, with the highest firearm-related homicide rates in Saskatchewan and Manitoba.^1^,^3^

Numerous individuals, including children and youth, present to hospital each year for unintentional firearm injuries.^4^ Between 2002 and 2016, for every firearm death, three people were treated for nonfatal injuries in Ontario hospitals.^5^

After the 2020 mass shooting in Nova Scotia, the deadliest mass shooting in Canada, the Mass Casualty Commission called for a cultural shift to institute a public health approach to firearm safety.^6^ A public health approach evaluates firearm access and the societal factors that contribute to or mitigate firearm violence. The Mass Casualty Commission emphasized that collaborating to address root causes such as the social determinants of health and deeply entrenched sociopolitical factors is essential for community safety.^6^ The Commission highlighted that resource allocation should focus on policing and law enforcement and on prevention; made numerous recommendations for regulations aimed at risk reduction; and emphasized the need for gender analysis, recognizing the effect of toxic masculinity among gun owners and the police.^6^

The design, implementation and effectiveness of any new firearm-injury prevention program relies on access to linked high-quality health, firearm, social and criminal justice data. At the time of writing, Canada lacks such firearm-related data because of funding constraints, political sensitivities, privacy concerns and other factors. The literature often highlights the need for qualitative data on the social determinants of firearm-related injuries and deaths as well as quantitative data on vulnerable populations most affected by firearm violence.^4^-^6^

Addressing these gaps is crucial because effective injury prevention strategies need to be informed by the social contexts and disparities that shape firearm risks. Successful injury prevention programs must involve community groups, victims, researchers, health care professionals, law enforcement personnel, policy-makers and other involved parties to ensure a comprehensive and collaborative approach.

To frame the impact of firearms as a public health issue, Canada needs a coordinated national effort and robust research initiatives. In this article, we aim to lay the groundwork for a national research agenda by identifying knowledge gaps in firearm-related injury and death and research priorities. 

We focused on three areas: unintentional firearm injuries; intimate partner violence (IPV) and femicide; and other assault-related firearm injuries. Unintentional firearm injuries are accidental and can be self-inflicted or inflicted by others.^7^ These injuries are often due to the mishandling, improper storage or unintended discharge of firearms.^8^ Assault-related firearm injuries, on the other hand, are intentional and violent.^8^ Injuries as a result of IPV and femicide are a distinct form of assault related to gender-based violence. We examined IPV/femicide-related firearm injury separately to capture the unique dynamics of this issue as well as the specialized support services and nuanced policy responses required. We did not examine firearm-related self-harm because of the broad scope of this topic and the different prevention priorities.

## Methods

We used nominal group technique, led by a certified professional facilitator, in two successive stages to achieve consensus on topics relevant to a national firearm research agenda (Table 1). 

**Table 1:** Stages of research

Stage	Objective	Study sample	Data collection tool	Analysis
1.	To identify current research needs and gaps	Key experts (N = 15)	Jamboard (digital whiteboard)	Document analysis and deductive content analysis
2.	To rank knowledge gaps	Canadian Academy of Health Sciences forum attendees (N = 43)	Feedback Frames^9^ (voting tool)	Quantitative analysis of scores after participant voting

The Unity Health Toronto research ethics board confirmed that this project did not require ethics approval because it does not constitute human research or contain any identifiable personal information.

***Participant recruitment***

Engaging a variety of people with broad interests is critical for identifying current needs in firearm research and providing diverse perspectives and expertise. Purposive sampling methods were used to recruit participants for both stages. Email invitations were sent to 36 key professional contacts with expertise in firearm injury and death in Canada between 24 July and 14 September 2023. These included experts in clinical practice and research in firearm injury, advocates for and leaders in violence prevention, decision-makers, individuals with lived experience (either directly or through loved ones) and community representatives. Specific groups included the Danforth Families for Safe Communities, an organization of community members affected by the 2018 shooting on Danforth Avenue, Toronto, Ontario, and YouthLink, which works with youth and families to improve community well-being.

***Stage 1: Identifying knowledge gaps***

Fifteen participants (Table 2) met virtually on 20 September 2023 to generate ideas on current knowledge gaps. They were placed into preselected breakout groups to ensure multidisciplinary and varied geographical representation. 

**Table 2:** Stage 1 participant characteristics (N = 15)

Characteristics	Size of sample, n
Female sex	12
Provinces represented	3
Professional role	
Professor	1
Advocacy group	5
Chief Firearms Officer	3
Epidemiologist	1
Physician	2
Other	3

All the discussions were conducted in English.

With the end goal to identify knowledge gaps in firearm-related injury and death in Canada, the focus groups were asked the following questions, which had been preselected by the research team: (1) What are the current research needs and gaps in unintentional firearm injury? (2) What are the current research needs and gaps in assault-related firearm injury? (3) What are the current research needs and gaps in intimate partner firearm injury/femicide? 

An interactive online whiteboard tool, Jamboard (Google, Mountain View, CA, US), was used to record responses.

The participants were then asked to pick those generated ideas that were most likely to lead to research projects based on two criteria, importance and feasibility. Importance refers to a project providing relevant and valuable insights; feasibility refers to the realistic implementation of a project using existing data or feasible novel data sources. The priority knowledge gaps were finalized after a facilitated group discussion that used nominal group technique to reach agreement. 

After the first meeting, the recorded knowledge gaps were rewritten in more formal language for review in Stage 2 (Table 3). 

**Table 3:** Summary of knowledge gaps selected at Stage 1

**Knowledge gaps in unintentional firearm injury (n = 5)**
The relationship between mental health history reported during firearm licence applications and mental health service utilization. The differential health and community impact of firearm injuries and deaths in rural vs. urban areas. The utilization and enforcement of child access protection laws after unintentional firearm injuries. The mental health service utilization patterns of family members after firearm injuries. The environment that led to unintentional firearm injuries: How was the firearm accessed? How were the firearm and ammunition stored? Did the injury happen at home?
**Knowledge gaps in assault-related firearm injury (n = 7)**
The risk factors that put people in situations that result in the injuries; proactively address/mitigate the risk to individuals. The economic cost of firearm injuries to victims’ families, community and Canadian society. A description of the available and required Canadian firearm-injury data, including impediments to data collection and use. The impact of assault-related firearm injuries on communities and the approaches to healing after such incidents. Canada-specific definitions and language around the different types and intents of firearm injuries, as well as their consequences. The relationship between social media and assault-related firearm injuries. The available and required support for individuals and/or their families after a firearm-related injury or death.
**Knowledge gaps in intimate partner firearm injury/femicide (n = 3)**
The available and required support for individuals and/or their families after a firearm-related injury or death in situations of IPV or femicide. The impact of social policies and legislation aimed at reducing IPV/femicide-related firearm injuries and death. Early indicators of or risk factors for IPV or femicide before the involvement of a firearm.

**Abbreviation:** IPV, intimate partner violence. 

***Stage 2: Ranking knowledge gaps***

Stage 2 took place at the Canadian Academy of Health Sciences forum, “Gun Violence is a Public Health Issue,” in Toronto, Ontario. The forum was open to all interested individuals; attendees included practitioners in community settings, representatives of the legal community, social workers, representatives from victim and survivor groups, and researchers in academia. The Stage 2 exercise and instructions were explained to all the forum attendees (approximately 150), who were encouraged to participate during breaks between presentations. The exercise occurred on 27 and 28 September 2023. The meetings and materials were in English.

The aim of Stage 2 was to rank the knowledge gaps selected in Stage 1. Using Feedback Frames,^9^ each of the 43 participants voted anonymously by dropping tokens assigned to different knowledge gaps into slots indicating “top priority,” “high priority,” “medium priority,” “low priority,” “disagree that it is a priority” and “not sure.” Also provided was an area to write comments about each knowledge gap. 

Votes were counted and quantitatively analyzed by the facilitator. Final scores to determine ranking were calculated using weighted scores (top priority = 5 points; high priority = 4 points; medium priority = 3 points; low priority = 2 points; “not sure” = 1 point; and “disagree that it is a priority” = 0 points). 

The following was used to calculate the numerical scores:

(5 # of Top priority tokens) + (4 # of High priority tokens) + (3 # of Medium priority tokens) + (2 # of Low priority tokens) + (1 # of Not sure tokens) + (0 # of Disagree priority tokens)

Total number of tokens

These numerical scores were then placed in order from highest to lowest to assign rank. The rank difference between two priorities was calculated as the difference between their scores.

## Results

***Research idea generation***

During Stage 1, the 15 participants generated 22, 16 and 33 free-text responses to the questions on the current research needs and gaps in unintentional firearm injury, intimate partner firearm injury/femicide and other assault-related firearm injuries, respectively (Figure 1).

**Figure 1A:**
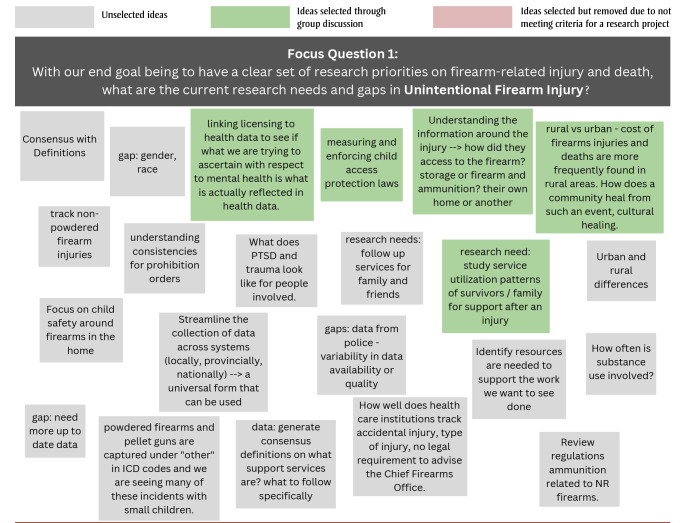
Free-text responses to Stage 1 focus question 1: What are the current research needs and gaps in unintentional firearm injury?

**Figure 1B:**
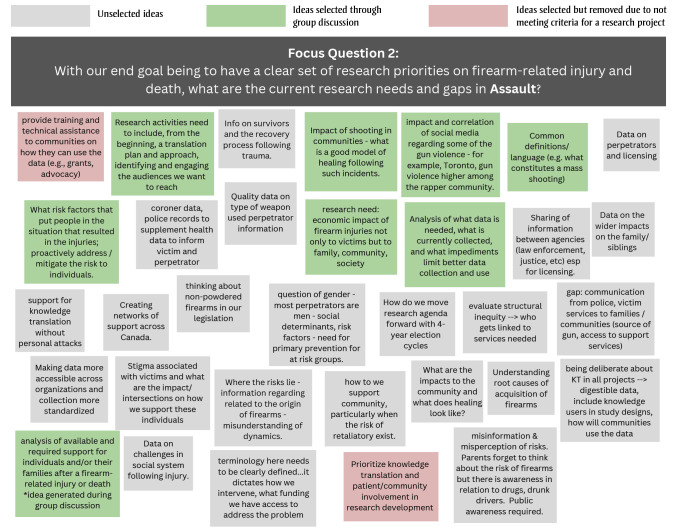
Free-text responses to Stage 1 focus question 2: What are the current research needs and gaps in assault-related firearm injury?

**Figure 1C:**
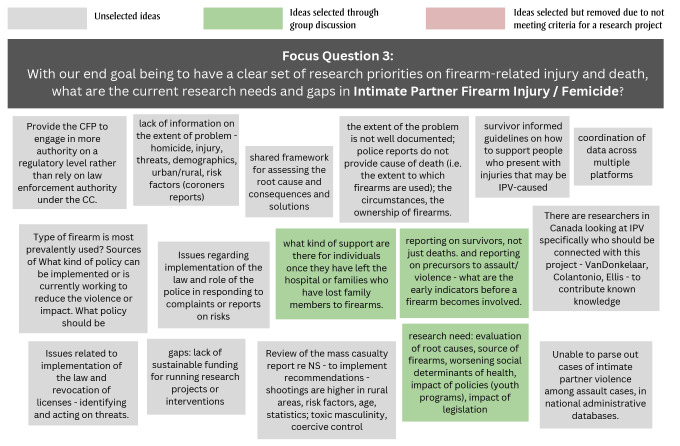
Free-text responses to Stage 1 focus question 2: What are the current research needs and gaps in assault-related firearm injury?

During subsequent facilitated discussions, the participants agreed on which research projects would be the most feasible and important. Across the three focus questions, 16 topics were selected; two were eliminated as they referred to knowledge translation rather than a research need or gap. Another knowledge gap, “the available and required support for individuals and/or their families after a firearm-related injury or death,” was generated and added during the facilitated group discussion. A total of 15 knowledge gaps were put forward for voting in Stage 2 (Table 3).

***Knowledge gap ranking***

The Stage 2 participants (N = 43) voted on the knowledge gaps using Feedback Frames. Participants did not vote on all 15 knowledge gaps; however, each station received votes from at least 38 individuals.

The highest-ranking knowledge gap, with a weighted average score of 4.1, was “the economic cost of firearm injuries to victims’ families, community and Canadian society” (Table 4). The next two highest-ranking priorities were “the impact of social policies and legislation aimed at reducing IPV- or femicide-related firearm injuries and death,” with a weighted average score of 4.0, followed by “a description of the available and required Canadian firearm-injury data, including impediments to data collection and use,” with an average score of 3.8. 

**Table 4:** Ranking results for knowledge gaps (n = 15)

Rank	Knowledge gaps	Number of votes, n						Weighted average score
		Top priority	High priority	Medium priority	Low priority	Disagree^a^	Not sure	
1	The economic cost of firearm injuries to victims’ families, community and Canadian society.	18	9	12	1	0	0	4.1
2	The impact of social policies and legislation aimed at reducing IPV/femicide-related firearm injuries and death.	13	16	9	2	0	0	4.0
3	A description of the available and required Canadian firearm-injury data, including impediments to data collection and use.	15	11	11	4	0	1	3.8
4	The available and required support for individuals and/or their families after a firearm-related injury or death.	8	13	17	1	0	0	3.7
5	Risk factors that put people in situations that result in injuries; proactively address/mitigate the risk to individuals.	7	20	10	4	0	1	3.7
6	The mental health service utilization patterns of family members after firearm injuries.	5	20	11	4	0	0	3.7
7	Early indicators of or risk factors for IPV or femicide before the involvement of a firearm.	9	12	18	3	0	1	3.6
8	The available and required support for individuals and/or their families after a firearm-related injury or death in situations of IPV or femicide.	6	16	17	3	0	0	3.6
9	The impact of assault-related firearm injuries on their communities and the approaches to healing after such incidents.	7	12	15	4	0	1	3.5
10	The relationship between social media and assault-related firearm injuries.	6	14	15	7	0	0	3.5
11	The differential health and community impact of firearm injuries and deaths in rural vs. urban areas.	6	14	12	7	0	0	3.5
12	The environment that led to unintentional firearm injuries: How was the firearm accessed? How were the firearm and ammunition stored? Did the injury happen at home?	7	10	10	9	1	1	3.3
13	The relationship between mental health history reported during firearm licence applications and mental health service utilization.	4	10	14	11	1	1	3.0
14	The utilization and enforcement of child access protection laws after unintentional firearm injuries.	5	5	16	8	1	3	2.9
15	Canada-specific definitions and language around the different types and intents of firearm injuries, as well as their consequences.	7	3	13	12	2	3	2.8

**Abbreviation:** IPV, intimate partner violence.

^a^ “Disagree that this [knowledge gap] is a priority.” 

Of note, the second-ranked research priority, the impact of social policies and legislation aimed at reducing IPV- and femicide-related firearm injuries and death, garnered the most top- and high-priority votes in total (n= 29), more than even the highest-ranked priority (n = 27), “the economic cost of firearm injuries to victims’ families, community and Canadian society.” Two research priorities that scored lower in the overall ranking also stood out because they received 25 or more votes categorizing them as a top or high priority: “risk factors that put people in the situations that result in the injuries; proactively address/mitigate the risk to individuals,” which ranked sixth (with 7top- and 20 high-priority votes) and “the mental health service utilization patterns of family members after firearm injuries,” which ranked eighth (5 top- and 20 high-priority votes).

Although the facilitator organized the Feedback Frames for ranking in such a way as to avoid choice overload and vote-splitting effects, there were two instances where ballot stuffing may have occurred, as more vote tokens were counted than the number of participant signatures for that station. This may have occurred because participants forgot to sign in. Regardless, these two research priorities ranked twelfth and thirteenth out of 15.

## Discussion

The 15 identified knowledge gaps offer valuable insights into areas on which to focus Canadian research efforts. Many of these priorities resemble the ones the National Research Council identified in 2013 in response to executive orders to learn about firearm violence in the United States.^10^ We did not identify any priorities related to gun safety technology or assessment of prevention strategies, however, likely due to a lack of substantial research in firearm safety technology and a scarcity of long-standing Canadian prevention programs.^6^

**Figure 2:**
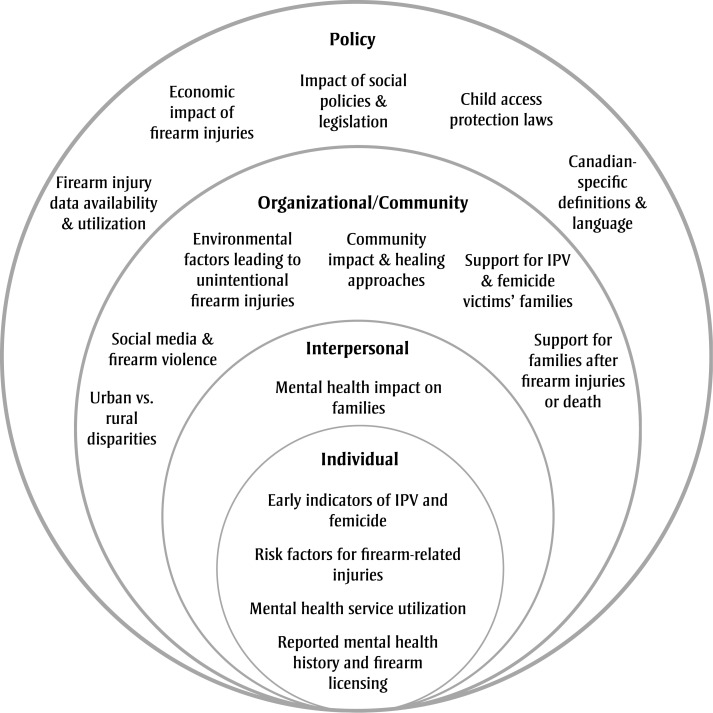
Social ecological model of firearm injury knowledge gaps

We identified themes from across the three categories—unintentional firearm injury, assault-related firearm injury and intimate partner firearm injury/femicide—that emphasize broader gaps such as risk factors and the overall impact of firearm injuries and deaths in Canada. To better organize these gaps and align with public health frameworks, we used a social ecological model that highlights research priorities at different levels, clarifying where knowledge gaps exist (Figure 2).^11^ We identified knowledge gaps at all levels of the model, emphasizing the need for comprehensive interventions that span individual, interpersonal, community and policy domains.

The knowledge gap selected as the top area to investigate was the economic cost of firearm injuries to victims’ families, communities and Canadian society. Detailed analyses of the current economic burden of firearm injuries could influence legislative change and improve funding of prevention programs. The most recent report on the economic and social impacts of firearm-related crime in Canada, in 2008, estimated the costs at more than CAD 3.1 billion;^12^ however, this was likely an underestimate as the consequences of suicide or unintentional firearm injuries and deaths were not taken into account.^12^

The second-ranked research priority was the impact of policies and legislation to reduce IPV- and femicide-related firearm injuries and death. Previous research on the effects of policies and laws on firearm injury and death rates had mixed results, with some studies suggesting that changes were significant and others reporting minimal effects.^13^-^15^ Further evaluation of, for example, *An Act to amend certain Acts and to make certain consequential amendments (firearms) *(the former Bill C-21) could help assess the influence of such legislation on firearm-injury rates among IPV victims.^16^

The third-ranked research priority focuses on describing the firearm-injury data that are available and that are required. A 2019 scoping review of empirical research on firearms in Canada identified only 34 peer-reviewed publications over 18 years.^13^ The available data are limited and fragmented, with siloed municipal, provincial/territorial, federal agencies and academic institutions collecting data according to their specific needs.^17^ These datasets are often inaccessible to other agencies or researchers, even though firearm-related data collected by, for example, the Ontario Ministry of Children, Community and Social Services during client assessments, could provide other agencies with valuable insights.^17^ Comparing data across regions is also hampered by inconsistent data collection methods and by the use of definitions that are neither standardized nor designed for diverse purposes.^17^

A summary of available data would help identify areas for standardization, facilitating future analyses of data from different agencies and institutions. For instance, firearms-related offences are more likely to involve multiple victims; however, some data systems input these as single incidents while others count these multiple times.^3^

In the last 3 years, the Canadian Association of Chiefs of Police and the Canadian Centre for Justice and Community Safety Statistics have worked to improve the national Uniform Crime Reporting Survey, which is used to measure the incidence of crime in Canada.^18^ Standard definitions of what constitutes a “shooting” and “crime gun” were added to the survey as recently as 2021.^18^ However, data on demographic variables such as victim and offender characteristics are still lacking.^13^,^18^ Collecting these data sensitively, by working with communities and with policing agencies, is critical to developing community-level public health interventions.^19^ By developing a central database that compiles all available firearm data from various agencies, we can continue to identify gaps and improve data collection.

Participants’ comments on lower-ranked research priorities included concerns about the feasibility of projects due to data privacy restrictions. For example, determining the relationship between mental health history reported during firearm licence applications and mental health service utilization was considered unfeasible due to a lack of linked data sources. Nevertheless, more than half of the participants considered risk factors in firearm events a top or high priority, and risk factor–related priorities ranked fifth and seventh out of the 15 knowledge gaps. Others commented that for specific priorities, the issues lie in the gaps between knowledge and actionability, and not in a lack of data.

Future work will focus on turning the highest priority knowledge gaps into actionable research projects. We will continue to work with interested parties and multidisciplinary groups to discuss data sources, methodological approaches and strategies to mitigate limitations.

***Limitations***

The findings in this report are subject to several limitations. 

Participants from only three provinces (Ontario, New Brunswick and Nova Scotia) took part in generated ideas during Stage 1. As such, their priorities may not reflect national or other provinces’ priorities. To help mitigate this, we gathered a team of diverse and multidisciplinary experts familiar with the issue of firearm-related injury on a national scale to take part in a rich discussion on knowledge gaps that covered many topics.

The potential for an “echo chamber” effect nevertheless existed as not all relevant perspectives were included in discussions, potentially limiting the diversity of ideas generated. For example, the predominance of female participants in Stage 1 may have influenced the prioritization of certain topics, reflecting perspectives more commonly emphasized by this demographic. Also, while the participants had many different public health and advocacy roles, the viewpoints of people working in economics, law enforcement or politics were not represented. Including a greater number of perspectives was beyond the feasibility of this project but would be valuable in future work.

There was potential for expertise bias because idea generation took place in a group setting, which may have allowed more assertive participants to influence the group. We minimized this effect by having multiple breakout groups and having a professional facilitator who elicited contributions from everyone. 

We decided on three focus questions to stimulate the generation of idea, but having more questions may have uncovered additional topics. 

Participants asked for clarifications and additional definitions regarding some of the priorities. Providing more detailed explanations about the knowledge gaps might have affected the way these participants ranked them. 

Lastly, as participant involvement occurred during the breaks between presentations at the “Gun Violence is a Public Health Issue” forum, the high participant traffic at these times may have led to a bandwagon effect, either through participants overhearing open discussions or seeing other participants’ actions despite efforts to make voting anonymous and conceal the results.

## Conclusion

With this study, we identified knowledge gaps related to firearm-related injury and death and research priorities that could inform a national research agenda for Canada. The top priorities highlight the large and diverse research gaps around firearm-related injuries and deaths. Next steps include operationalization of these top knowledge gaps into research questions, identification of data sources and optimal methodological approaches, and decision-making regarding knowledge translation strategies. The results of this consultation process may serve as a catalyst to create impactful and much-needed firearm-related priority prevention research.

## Acknowledgements

We thank Karyn Dumble of Monarch Park Group, Toronto, Ontario, for her critical role as a certified professional facilitator. We also thank the experts who contributed their invaluable knowledge during the various phases of this work.

## Funding

This study was supported by a Canadian Institutes of Health Research (CIHR) Institute of Cancer Research (ICR) Planning and Dissemination Grant (Funding reference number PCS 183367).

## Conflicts of interest

None.

## Authors’ contributions and statement

LA: Formal analysis, writing—original draft.

AS, AY, CG, AB, CS, WC, IW, WT, ST, NB, BB, NS: Formal analysis, writing—review and editing.

DG: Conceptualization, methodology, investigation, project administration, supervision, formal analysis, writing—review and editing.

The content and views expressed in this article are those of the authors and do not necessarily reflect those of the Government of Canada.
